# Hospitalizations for Crohn’s Disease — United States, 2003–2013

**DOI:** 10.15585/mmwr.mm6614a1

**Published:** 2017-04-14

**Authors:** Christopher A. Malarcher, Anne G. Wheaton, Yong Liu, Sujay F. Greenlund, Suraj J. Greenlund, Hua Lu, Janet B. Croft

**Affiliations:** ^1^Oxford College of Emory University, Oxford, Georgia; ^2^Division of Population Health, National Center for Chronic Disease Prevention and Health Promotion, CDC; ^3^Georgia State University, Atlanta, Georgia; ^4^Georgia Institute of Technology, Atlanta Georgia.

In 2009, an estimated 565,000 Americans had Crohn’s disease (*1*), an inflammatory bowel disorder that can affect any part of the gastrointestinal tract. Symptoms include persistent diarrhea, abdominal cramps and pain, constipation leading to bowel obstruction, and rectal bleeding.* Symptoms sometimes intensify in severity and require hospitalization and surgeries of the small intestine, colon, or rectum (*2*). Hospital discharge data from the National Inpatient Sample (NIS) of the Healthcare Cost and Utilization Project (HCUP) were used to estimate U.S. hospitalizations^†^ for Crohn’s disease as both the first-listed and any-listed^§^ discharge diagnosis and common surgical procedures during hospitalizations with Crohn’s disease as first-listed diagnosis from 2003 to 2013, the most recent decade of data. Despite new therapies that were expected to improve remission and reduce hospitalizations, estimated numbers (and age-adjusted rates per 100,000 U.S. population) of hospitalizations for Crohn’s disease as the first-listed diagnosis did not change significantly from 2003 to 2013. The proportion of these hospitalizations during which small bowel resection was performed decreased from 4.9% in 2003 to 3.9% in 2013 (p<0.05); however, colorectal resection and fistula repair rates remained stable. Hospital stays for any-listed Crohn’s disease increased from >120,000 (44.2 per 100,000) in 2003 to >196,000 (59.7 per 100,000) in 2013 (p<0.05). Patient education initiatives should focus on increasing awareness of exacerbating factors and medication compliance to prevent hospitalizations.

NIS hospital discharge data, which were obtained from the Agency for Healthcare Research and Quality (AHRQ), represent an annual stratified sample of 7–8 million hospital records collected by 37–44 participating states from approximately 20% of U.S. community hospitals.^¶^ Records are weighted for hospital characteristics as well as for patient diagnoses, age, and admission month, so that analyses can produce reliable national estimates. Because the NIS implemented a new systematic sampling design in 2012, revised sampling weights were used for all analyses. Crohn’s disease was defined for a first-listed diagnosis field and for any of 15 diagnosis fields during 2003–2008 and 25 fields during 2009–2013 with *International Classification of Diseases, Ninth Revision, Clinical Modification* (ICD-9-CM) disease codes 555.0–555.9. For states, the number of hospitalizations in 2013 with any diagnosis of Crohn’s disease was obtained using the online query tool** from 35 states participating in the State Inpatient Databases, HCUP, AHRQ. Denominators for all annual hospitalization rates by age, sex, and state were obtained from the U.S. Census intercensal estimates for 2003–2009^††^ and 2010–2013.^§§^ Direct age-adjustment to the projected 2000 U.S. population was performed using five age groups (<18, 18–44, 45–64, 65–84, and ≥85 years) to calculate age-adjusted hospitalization rates per 100,000 U.S. population and 95% confidence intervals (CIs). Because race/ethnicity was not reported for 26% of hospital records in 2003 and 6% in 2013, these analyses do not include estimates by race/ethnicity. The percentages of first-listed hospitalizations that included small bowel resection, colorectal resection, or fistula repair were also obtained from any of up to 15 procedure fields.^¶¶^ SAS-callable SUDAAN was used to account for the complex sampling design of NIS. For comparisons of rates from 2003 to 2013 and between males and females, statistical significance (p<0.05) was determined by the Z-test.

Hospitalization rates for any-listed diagnosis of Crohn’s disease were two times higher than those for a first-listed diagnosis from 2003 to 2007 and three times higher from 2008 to 2013 (Figure 1). Potential reasons for the sharp increase in hospitalization rates from 2007 to 2008 are unknown; however, there were no changes in NIS methodology, states reporting data, or diagnosis codes at that time. Age-adjusted hospitalization rates were higher among females than among males for both first-listed and any-listed diagnosis of Crohn’s disease (p<0.05).

**FIGURE 1:**
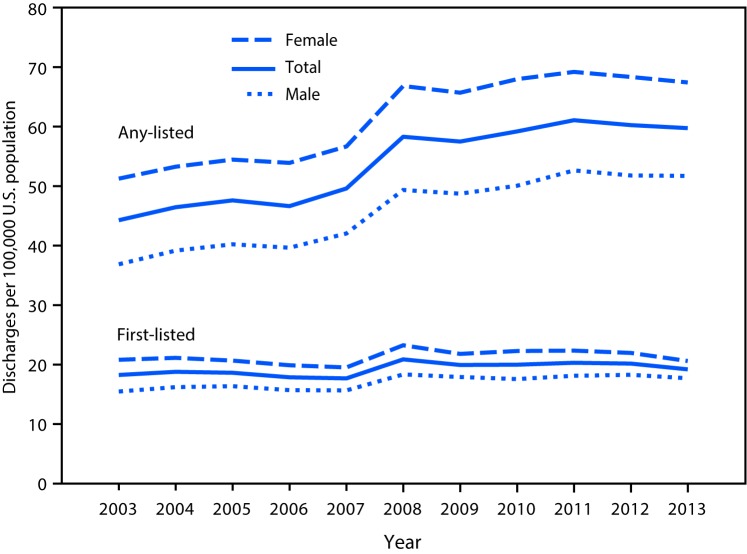
Age-adjusted* hospitalization rate (per 100,000 population) for a first-listed or any-listed diagnosis^†^ of Crohn’s disease, by sex — National Inpatient Sample, United States, 2003–2013 *Age-adjusted to the 2000 projected U.S. population. ^†^ First-listed Crohn’s disease diagnosis indicates that Crohn’s disease was the principal reason for the hospitalization. Any-listed Crohn’s disease diagnosis indicates that patients had Crohn’s disease, but Crohn’s disease was not necessarily the main reason they were being hospitalized.

From 2003 to 2013, there was no significant change in the estimated number of hospitalizations for Crohn’s disease as a first-listed diagnosis; however the age-adjusted hospitalization rate for Crohn’s disease as any-listed diagnosis increased 35.1% from 44.2 per 100,000 (120,209 hospitalizations) in 2003 to 59.7 per 100,000 (196,480 hospitalizations) in 2013 (Table). As a first-listed diagnosis, there was a significant increase in the hospitalization rate in 2013 relative to 2003 (+14.5%), only among males (p<0.05). In contrast, hospitalization rates for any-listed Crohn’s disease increased from 2003 to 2013 for all groups defined by age and sex. In both 2003 and 2013, hospitalization rates for Crohn’s disease as a first-listed diagnosis were higher among persons aged 18–44 years than among other age groups, whereas, hospitalization rates with any-listed Crohn’s disease increased among successive age groups until ages 65–84 years.

**TABLE:** Hospitalizations for Crohn’s disease, by selected characteristics — National Inpatient Sample, United States, 2003 and 2013

Characteristic	2003		2013		Relative change (%)^§^
	No.*	Hospitalization rate (95% CI)^†^	No.*	Hospitalization rate (95% CI)^†^	
**First-listed diagnosis**					
**Total**					
Unadjusted	52,855	18.2 (16.4–20.0)	60,255	19.1 (18.3–19.8)	+4.6
Age-adjusted		18.2 (16.8–19.5)		19.1 (18.5–19.7)	+5.2
**Age group (yrs)**					
<18	3,384	4.6 (3.4–5.8)	4,410	6.0 (5.0–7.0)	+29.4
18–44	29,666	26.4 (23.4–29.4)	33,149	28.9 (27.5–30.2)	+9.4
45–64	13,938	20.2 (18.2–22.3)	15,745	19.0 (18.0–19.9)	−6.4
65–84	5,333	17.0 (15.3–18.7)	6,370	16.5 (15.5–17.5)	−3.0
≥85	533	11.9 (9.3–14.6)	580	9.6 (7.8–11.4)	−19.6
**Sex**					
Male	21,931	15.4 (14.1–16.6)	27,330	17.6 (16.9–18.3)	+14.5^¶^
Female	30,751	20.7 (19.2–22.2)	32,920	20.5 (19.7–21.3)	−1.0
**Any-listed diagnosis**					
**Total**					
Unadjusted	120,209	44.5 (41.1–47.9)	196,480	62.1 (60.1–64.2)	+39.5^¶^
Age-adjusted		44.2 (41.9–46.5)		59.7 (58.3–61.1)	+35.1^¶^
**Age group (yrs)**					
<18	5,005	6.8 (5.2–8.5)	7,115	9.7 (8.2–11.2)	+41.2^¶^
18–44	56,303	50.1 (45.3–54.9)	77,220	67.3 (64.4–70.2)	+34.2^¶^
45–64	41,144	59.8 (55.3–64.2)	63,175	76.0 (73.2–78.8)	+27.0^¶^
65–84	23,666	75.4 (69.8–81.0)	42,495	109.9 (105.9–113.9)	+45.8^¶^
≥85	3,090	69.2 (60.9–77.5)	6,475	107.2 (100.1–114.3)	+54.9^¶^
**Sex**					
Male	51,454	36.8 (34.7–38.9)	82,035	51.7 (50.3–53.1)	+40.5^¶^
Female	77,514	51.2 (48.5–53.9)	114,425	67.4 (65.7–69.1)	+31.6^¶^

**Abbreviation:** CI = confidence interval.

* Estimated number of hospitalizations.

^†^ Hospitalization rate (per 100,000 U.S. population) and 95% CI. Age-adjusted to the 2000 projected U.S. population, except for age groups.

^§^ Change in 2013 relative to 2003.

^¶^ Statistically significant difference (p<0.05) in rates from 2003 to 2013 using the Z-statistic.

Geographic variations were observed in tertiles of age-adjusted hospitalization rates with any-listed Crohn’s disease in 2013 among participating HCUP states (Figure 2), with age-adjusted hospitalization rates per 100,000 ranging from 19.2 in Hawaii to 91.6 in Rhode Island. States with the lowest hospitalization rates were clustered in the Southwest and Rocky Mountain states.

**FIGURE 2:**
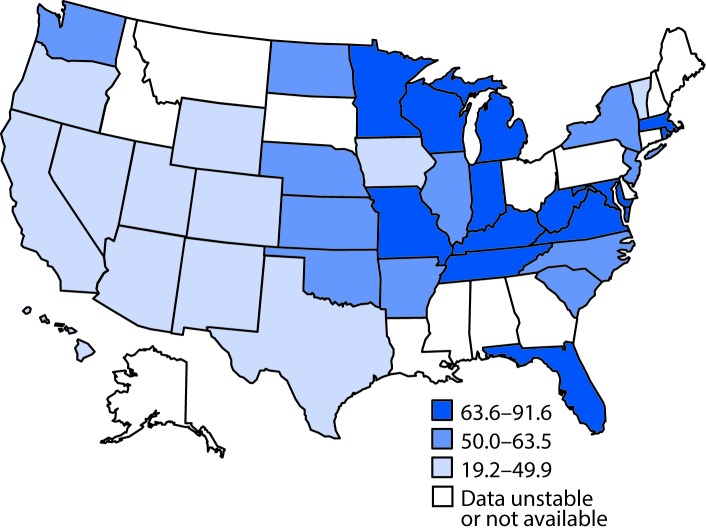
Age-adjusted* hospitalization rate^†^ (per 100,000 population) for any-listed diagnosis^§^ of Crohn’s disease — State Inpatient Databases, 2013 * Age-adjusted to the 2000 projected U.S. population. ^†^ Estimates considered unstable if relative standard error >30%. ^§^ Any-listed Crohn’s disease diagnosis indicates that patients had Crohn’s disease, but Crohn’s disease was not necessarily the main reason they were being hospitalized.

Among hospitalizations for a first-listed diagnosis of Crohn’s disease in 2013, 3.9% were for small bowel resection, 12.8% for colorectal resection, and 2.0% for fistula repair. The decline in the percentage of hospitalizations for small bowel resection from 2003 (4.9%) was significant (p<0.05), but the percentages of 2003 hospitalizations for colorectal resections (14.8%) or fistula repairs (1.8%) were similar to those in 2013.

## Discussion

Stable trends for a first-listed Crohn’s disease diagnosis from 2003 to 2013 suggest that the 4.3% annual increase reported for a first-listed diagnosis from 1998 to 2004 (*3*) has not continued. Although this result suggests that the available treatments have not increased clinical remissions or reduced hospitalizations, it is possible that these trends indicate the beginning of a reversal of the increased hospitalizations and surgical procedures observed in the years leading up to the study period. The proportion of hospitalizations with small bowel resection declined from 2003 to 2013, with no significant change in colorectal resections and fistula repairs. These trends contrast with the period from 1993 to 2004, when rates of small bowel and right colon resection did not change and fistula repairs increased significantly (*4*). Resections are only performed on an inpatient basis and the decline cannot be explained by increases in outpatient procedures; therefore, the declines in small bowel resection during hospitalizations might represent a decrease in clinical severity, possibly related to newer therapies. Hospitalizations with any-listed diagnosis of Crohn’s disease continued to increase in the most recent decade. This increase might represent greater physician awareness and diagnosis of Crohn’s disease or more complete coding of secondary diagnoses by physicians.

State variations in any-listed hospitalization for Crohn’s disease are similar to findings in earlier reports showing state-specific first- and any-listed hospitalization rates during 2001–2006 (*5*). Previous studies found lower first-listed hospitalization rates in western U.S. regions during 1998–2004 (*3*), lower prevalence of Crohn’s disease among insured adults in the South and West, and higher prevalence in the Midwest during 2008–2009 (*1*). Whether this consistent regional pattern is the result of variations in physician awareness and diagnosis of Crohn’s disease or variations in risk factors for Crohn’s disease is unknown.

The findings in this report are subject to at least five limitations. First, information on hospital diagnosis and procedures is based solely on ICD-9-CM codes that are reported in the hospital record, which cannot be validated in this study and should not be interpreted as new incident cases. Second, severity of the condition cannot be determined for most cases, other than that surgical procedures were performed during some hospitalizations. Third, the surveillance estimates for surgical procedures in this study only represent hospital inpatient records and do not include procedures performed in outpatient clinics, or small bowel resections and colon resections conducted for first-listed diagnosis codes other than Crohn’s disease. It is also likely that the number of hospital discharges for Crohn’s disease includes an undetermined number of repeat hospital stays for some persons. Fourth, although the reporting of race/ethnicity on hospital records has improved during the past decade, the continued absence of such sociodemographic information from a significant proportion of records constrains assessment of possible disparities in this low prevalence chronic disease. Finally, the lack of data for every state limits the ability to detect geographic clustering of hospital discharge rates.

Diagnosis of Crohn’s disease is based on a combination of gastrointestinal endoscopy, imaging, and pathologic studies (*2*). Progression of treatment for a patient usually proceeds from aminosalicylates and corticosteroids to immunomodulators and other biologic therapies, and to surgery in severe cases (*2*). From 1994 to 2005, prescriptions declined for corticosteroids and increased for immunomodulatory or biologic therapies during office visits involving a diagnosis of either Crohn’s disease or ulcerative colitis (*6*).

Risk factors for Crohn’s disease are not clearly established. Cigarette smoking has been suggested as a risk factor and is recognized to increase disease severity among patients with Crohn’s disease (*7*). Upper respiratory or enteric infections, nonsteroidal anti-inflammatory drugs, and possibly stress might initiate and exacerbate symptoms and lead to hospitalizations (*2*). A major deterrent in identifying risk factors is that Crohn’s disease is assumed to be a low prevalence chronic disease. Large sample size studies, which would be prohibitively expensive, would be required for national surveillance systems to collect reliable information on the prevalence of existing cases and the incidence of new cases in the general U.S. population and in subgroups, such as children and minorities (*8*).

Because the cause of Crohn’s disease is unknown, it is difficult to determine what changes in public health practice could help prevent it. Patient education initiatives could focus on increasing awareness of exacerbating factors such as cigarette smoking and stress, and medication compliance to prevent hospitalizations. Professional education should continue to increase awareness of the signs and symptoms of Crohn’s disease and improve diagnosis and management.

**Summary**

**What is already known about this topic?**

Hospital discharges for a first-listed diagnosis of Crohn’s disease increased from 1993 to 2004, despite new therapies that were expected to improve remission and reduce hospitalizations.

**What is added by this report?**

Hospitalizations for a first-listed diagnosis of Crohn’s disease did not change from 2003 to 2013. In addition, inpatient surgical procedures for small bowel resection declined, whereas those for colorectal resection or fistula repairs remained stable. It is unclear whether these trends indicate the beginning of a reversal of the increases in hospitalizations and surgical procedures observed in the years leading up to study period. The increase in hospitalizations for any-listed diagnosis of Crohn’s disease might represent greater physician awareness and diagnosis of the condition. State-specific estimates suggest geographic variation in hospitalizations.

**What are the implications for public health practice?**

Because the risk factors are not known, public health prevention programs are not possible. However, stress reduction and smoking cessation might be beneficial in ameliorating disease severity among patients with Crohn’s disease. Professional education should continue to increase awareness of the signs and symptoms of Crohn’s disease, and improve diagnosis and management of the disease.

## Footnotes

* http://www.crohnscolitisfoundation.org/what-are-crohns-and-colitis/what-is-crohns-disease.

† Hospitalizations or hospital stays refer to a patient being admitted to a hospital for one or more days.

§ First-listed Crohn’s disease diagnosis indicates that Crohn’s disease was the principal reason for the hospitalization. Any-listed Crohn’s disease diagnosis indicates that patients had Crohn’s disease, but Crohn’s disease was not necessarily the main reason they were being hospitalized.

¶
https://www.hcup-us.ahrq.gov/db/nation/nis/NISIntroduction2012.pdf .

** https://www.hcup-us.ahrq.gov/sidoverview.jsp. A list of the HCUP Data Partners that contribute to HCUP is available at https://www.hcup-us.ahrq.gov/db/hcupdatapartners.jsp.

†† Intercensal Estimates of the Resident Population by Sex and Age for the United States: April 1, 2000 to July 1, 2010. https://www2.census.gov/programs-surveys/popest/tables/2000-2010/intercensal/national/us-est00int-01.xls.

§§ Annual Estimates of the Resident Population: April 1, 2010 to July 1, 2014. 2014 Population Estimates. https://factfinder.census.gov/faces/tableservices/jsf/pages/productview.xhtml?pid=PEP_2016_PEPANNRES&src=pt.

¶¶ ICD-9-CM procedure codes: small bowel resection (45.61–45.63), colorectal resection (17.3, 45.71–45.79, 45.81–45.83, 48.40–48.49, 48.50–48.59, or 48.61–48.69), fistula repair (46.74, 46.76, 48.73, 48.93, 49.11, 49.12, 49.73, 57.83, 58.43, or 70.72–70.74).
